# A systematic approach to the recurrent laryngeal nerve dissection at the cricothyroid junction

**DOI:** 10.1186/s40463-018-0306-7

**Published:** 2018-09-17

**Authors:** Oleksandr Butskiy, Brent A. Chang, Kimberly Luu, Robert M. McKenzie, Donald W. Anderson

**Affiliations:** 1 Division of Otolaryngology – Head & Neck Surgery, University of British Columbia, Vancouver, BC Canada; 2 Gordon & Leslie Diamond Health Care Centre, 4th. Fl. 4299B-2775 Laurel Street, Vancouver, BC V5Z 1M9 Canada

**Keywords:** Surgical technique, Thyroidectomy, Recurrent laryngeal nerve, Retrograde dissection, Surgical anatomy

## Abstract

**Background:**

To describe and evaluate a four step systematic approach to dissecting the recurrent laryngeal nerve (RLN) starting at the cricothyroid junction during thyroid surgery (subsequently referred to as the retrograde medial approach).

**Methods:**

All thyroidectomies completed by the senior author between August 2014 and January 2016 were retrospectively reviewed. Patients were excluded if concurrent lateral or central neck dissection was performed. A follow up period of 1 year was included.

**Results:**

Surgical photographs and illustrations demonstrate the four steps in the retrograde medial approach to dissection of the RLN in thyroid surgery.

Three hundred forty-two consecutive thyroid surgeries were performed in 17 months, including 213 hemithyroidectomies, 91 total thyroidectomies, and 38 completion thyroidectomies. The rate of temporary and permanent hypocalcemia was 13% (95% confidence interval [CI]: 8–20%) and 3% (95% CI: 1–8%) respectively. The rate of temporary and permanent vocal cord palsy was 9% (95% CI: 6–12%) and 0.3% (95%CI: 0.01–2%) respectively. The median surgical times for hemithyroidectomy, total thyroidectomy, and completion thyroidectomy were 39 min (Interquartile range [IQR]: 33–47 min), 48 min (IQR: 40–60 min), and 40 min (IQR: 35–51 min) respectively. 1% of cases required conversion to an alternative surgical approach.

**Conclusion:**

In a tertiary endocrine head and neck practice, the routine use of the retrograde medial approach to RLN dissection is safe and results in a short operative time, and a low conversion rate to other RLN dissection approaches.

## Background

Thyroid surgeries are the most frequent operations performed by head and neck surgeons in the United States [1]. 2015 American Thyroid Association Guidelines recommend “Visual identification of the recurrent laryngeal [RLN] during dissection in all cases” on moderate-quality evidence [2]. Three approaches to RLN identification have been described: lateral approach, inferior approach, and the superior approach [3].

**Fig. 1:**
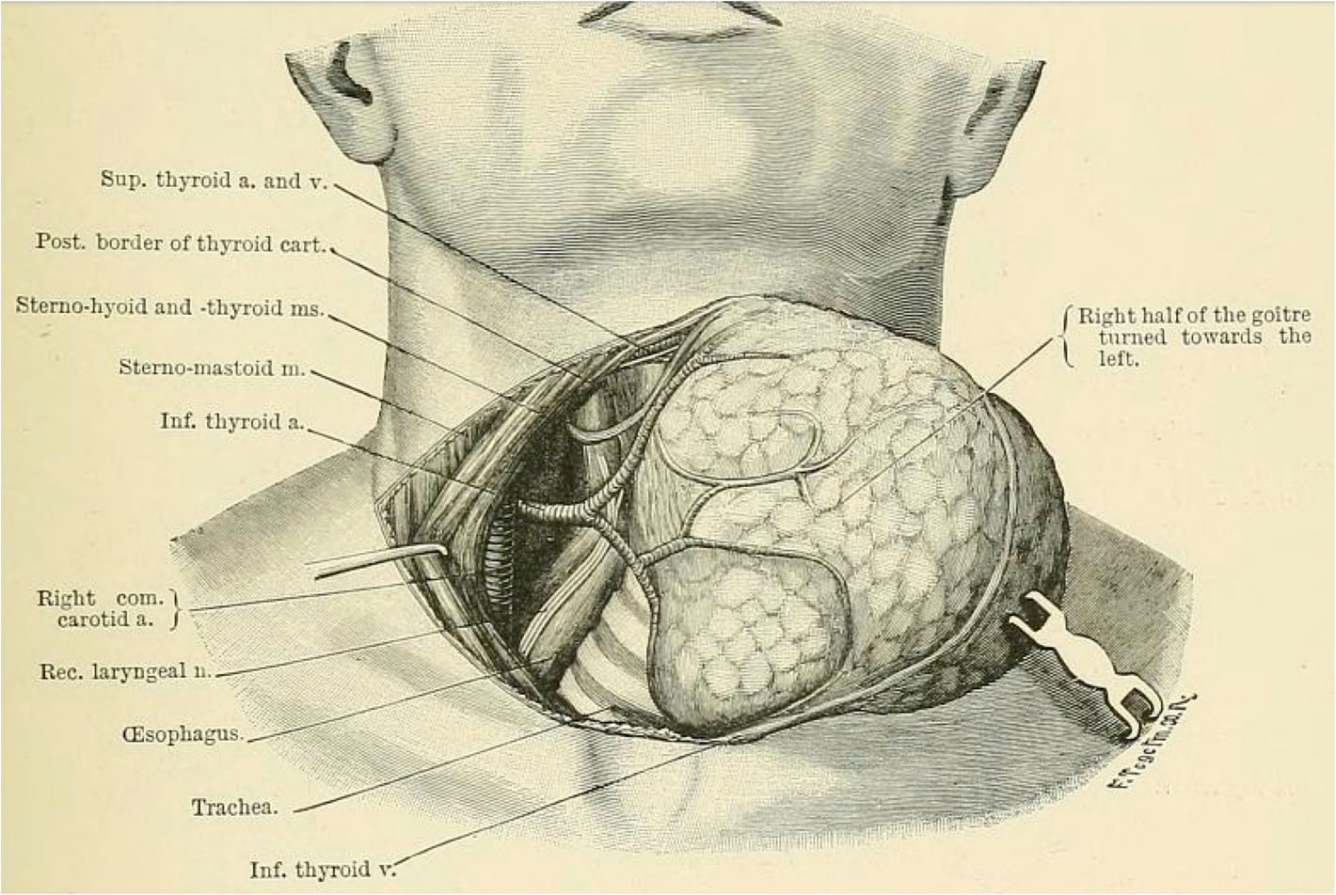
Illustration to Theodor Kocher’s 1895 surgical textbook demonstrating the dissection in the tracheo-esophageal groove after the thyroid is swept medially [4]

The lateral approach is routinely used by most surgeons for uncomplicated thyroid surgery and was advocated since the time of Theodor Kocher (Fig. 1) [4]. In this approach the thyroid lobe is retracted medially, middle thyroid vein is divided, and the RLN is identified at the mid-polar level.

For revision cases and for goiter surgery, the inferior approach is often used. In this approach the RLN is found in the soft areolar tissue in the tracheoesophageal groove proximal to the inferior thyroid artery crossing point. One advantage of this technique is that the RLN is found proximally prior to extra-laryngeal branching and away from thyroid bed scarring that might have been caused by prior surgery [3].

The superior Superior approach is the least used approach. In this approach, the RLN is identified as it enters under the inferior constrictor muscle proximal to the cricothyroid junction [3]. This approach is advantageous as the RLN position relative to the cricothyroid junction is consistent regardless of thyroid pathology and congenital variations [5–8].

The surgical technique for the superior approach has not been described in sufficient detail and its outcomes have not been documented well in literature. Only three studies report on the routine use of the superior approach: one study used a single sentence to describe the surgical technique [9], and the authors of the other two studies followed a technique that appears to be a modification of the lateral approach [6, 10]. In this technique the thyroid is retracted medially, and the RLN is found by searching between Zukerkandl’s tubercle and the cricopharyngeus muscle [6]. Thus, the RLN is not found right at the cricothyroid junction. While a method of identifying the RLN at the cricothyroid junction is likely known to experienced thyroid surgeons, this method has not been described in detail nor evaluated previously in literature.

The main objective of this study was to describe a four step standardized surgical technique to identifying the RLN at the cricothyroid junction (subsequently referred to as the retrograde medial approach). The primary outcome of interest  was the rate of surgical complications (temporary and permanent hypocalcemia and vocal cord palsy). The secondary outcomes were surgical time and conversion rate to other techniques.

## Methods

The University of British Columbia (UBC) Research Ethics Board granted approval (H15–01667) for the study. STROBE Statement for cohort studies was followed in reporting the study [11]. Study was designed as a retrospective review of a cohort of consecutive thyroidectomy patients. All eligible patients were referred to a tertiary head and neck surgical practice affiliated with the University of British Columbia (UBC) for consideration of a thyroidectomy.

All thyroidectomies were performed at teaching hospitals where residents assumed increasing responsibility to complete the procedure as their experience developed. The operations were performed between August 2014 and January 2016.

### Participants

All patients who underwent a hemi-, total, or completion thyroidectomy between August 2014 and January 2016 were selected. To obtain an estimate of the surgical time attributed solely to thyroid dissection, patients were excluded if concurrent lateral or central neck dissection was performed. Pre-operatively all patient underwent visual examination of the larynx, but PTH, Calcium and Vitamin D levels were not routinely measured.

### Post-operative care

After hemithyroidectomy, all patients were observed for 4 hours and were discharged home barring complications. Completion thyroidectomy and total thyroidectomy patients were admitted overnight and their ionized calcium level was checked in the morning. Patients were discharged if the ionized calcium level was above 1.00 mmol/L. Routine calcium/vitamin D supplementation and surgical drains were not used.

### Follow-up

Post-operative follow-up consisted of an office visit 2 weeks after the operation or earlier on patient request. Post-operative larynx visualization was performed only if patients described voice or swallowing abnormalities on specific questioning. If any abnormality was detected on the first post-operative visit, such as hypocalcemia or vocal cord palsy, the patients were followed monthly for up to a year or until the abnormality resolved.

### Data collection, variables and data sources

Four co-investigators (OB, BAC, RMM, and KL) collected data retrospectively from August 2016 to May 2017. Patient demographic and surgical indications data was obtained from clinic notes. Procedures performed, surgical times, and admission duration data were obtained from hospital records. Surgical time was defined as the time from first incision to wound closure completion as documented by the nursing staff.Operative dictations were used to establish if conversion to an alternative RLN dissection technique occurred. Pathological diagnoses and thyroid weights were determined reviewing the final pathology reports.

Data on the following complications were extracted from hospital and clinic records: temporary and permanent hypocalcemia and vocal cord palsy, hematoma, seroma, wound infection, and subcutaneous emphysema. Superior laryngeal neve palsy was not looked for during the follow up visit unless deemed necessary. Temporary hypocalcemia was defined as any hypocalcemia requiring calcium or vitamin D supplementation within 6 months of surgery. Permanent hypocalcemia was defined as any hypocalcemia requiring supplementation for greater than 6 months after surgery. Temporary vocal cord palsy was defined as the loss of true vocal cord adduction lasting less than 6 months of surgery, after which the palsy was deemed permanent. Hematoma was defined as any bleeding requiring reoperation or drain placement. In addition, the provincial health care database was screened for post-operative complications related hospital visits that might not have been captured in clinic charts. Patients with missing data were included in the study.

### Surgical technique

Skin incision, elevation of subplatysmal flaps, separation of strap muscles, and release of the sternothyroid muscles to expose the thyroid gland were performed in the usual manner. Unless invaded by the tumor, sternothyroid muscles were not cut. All vessel ligation was performed with Adson insulated bipolar forceps (Kirwan Surgical Products, Marshfield, MA) without routine use of suture ties or surgical clips. RLN monitor was not used. The next four steps are critical to the described technique.

1. Isolation and division of the isthmus:

The thyroid isthmus is isolated and divided over the trachea using bipolar cautery.

**Fig. 2:**
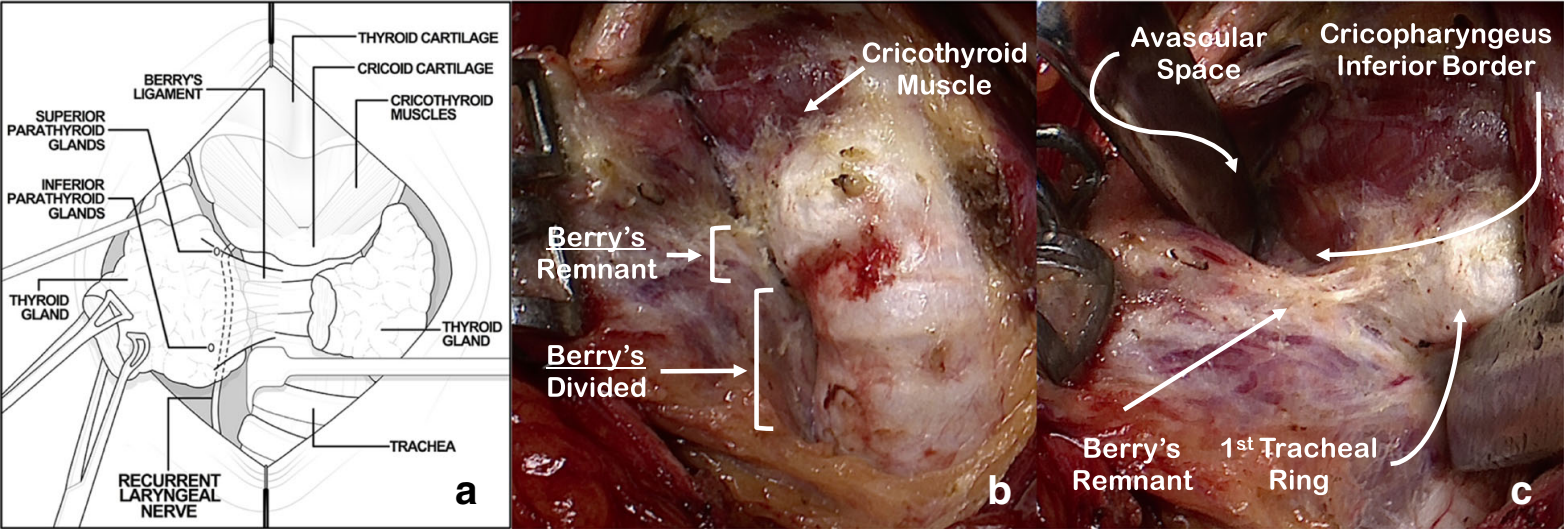
Subtotal division of Berry’s suspensory ligaments and exposure of the cricothyroid region. **a** Schematic demonstrating anatomy and instrument orientation; **b** Subtotal division of Berry’s ligaments; **c** Placing Berry’s ligament remnant on the stretch with avascular space open

2. Subtotal division of Berry’s suspensory ligaments and exposure of the cricothyroid region (Fig. 2):

The divided edge of the isthmus is grasped with Babcock clamps and retracted laterally (Fig. 2a). Inferior to the first tracheal ring, Berry’s suspensory ligaments are divided to separate the thyroid away from the trachea (Fig. 2b). Retraction of the trachea toward the contralateral side facilitates performance of this step. Care is taken to dissect directly on the trachea, as the RLN will be protected by a layer of fascia and ligamentous tissue. Lateral to the first tracheal ring the full thickness of Berry’s suspensory ligament is left intact (Fig. 2c).

The avascular space between the superior pole and the cricothyroid muscle is developed avoiding damage to the superior laryngeal nerve and the cricothyroid muscle (Fig. 2c). Differential traction between the superior pole and the trachea (demonstrated by the Langenbeck retractors in Fig. 2a & c) is critical for exposure and identification of the anatomical structures. Bowstring tension promotes isolation and identification of the Berry’s ligament remnant. Subsequent to injury-free identification of the cricothyroid muscle, care is then taken to expose the inferior border of the cricopharyngeus muscle, a critical landmark for RLN identification (Fig. 2c).

**Fig. 3:**
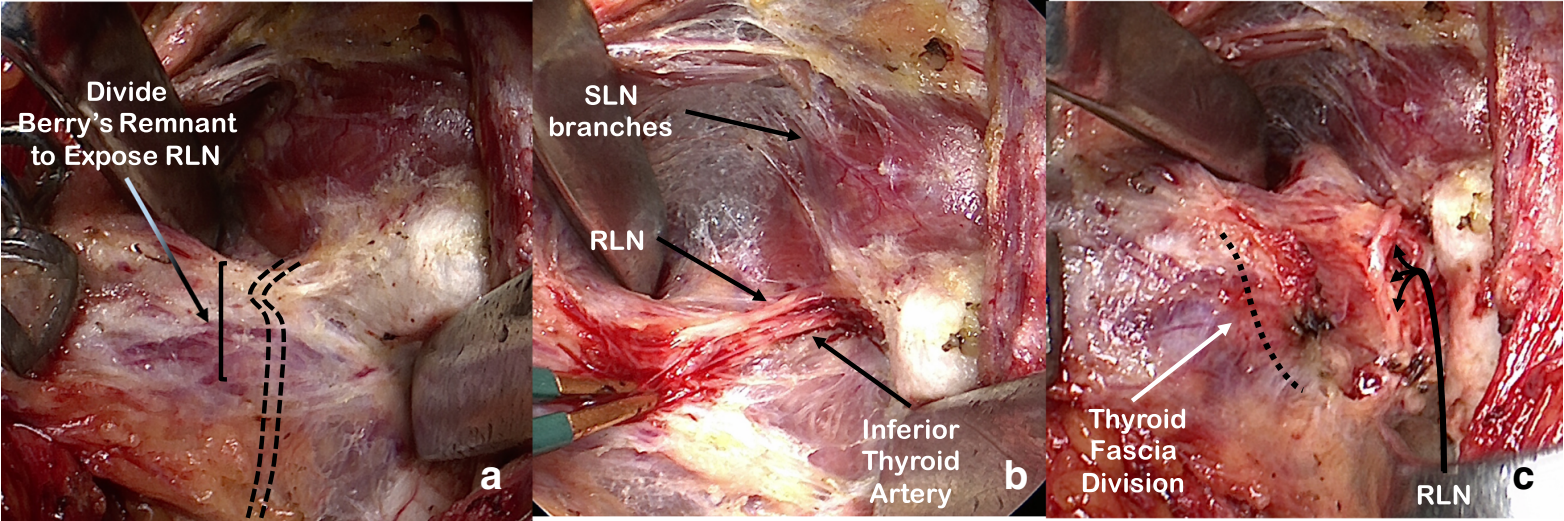
Identification and dissection of the recurrent laryngeal nerve (RLN). **a** RLN path deep to the Berry’s ligament remnant; **b** Berry’s ligament remnant dissected to reveal the RLN and the terminal branch of the inferior thyroid artery (nerve is placed on retraction to demonstrate anatomy to the photographer); **c** Thyroid fascia divided. Superior Laryngeal Nerve (SLN)

3. Identification and dissection of the recurrent laryngeal nerve (Fig. 3):

At this point, the remnant of the Berry’s ligament tethers the thyroid to the superior portion of the first tracheal ring and protects the RLN from the stretch of the retractors (Fig. 3a). Deep to the remnant are the RLN and the inferior thyroid artery terminal branches. The RLN runs in a plane posterior to Berry’s ligaments and then turns medially to enter the larynx at the superior aspect of the ligamentous tissue, forming an anatomical ‘genu’ [8]. Careful dissection through the superior portion of this ligamentous tissue using a fine mosquito forceps and bipolar cautery allows identification of the RLN insertion under the inferior constrictor (Fig. 2b). Inferior border of the cricopharyngeus muscle is visualized at all times and serves as a landmark for the depth at which the RLN is found. Dissection is facilitated by judicious and careful control of the inferior thyroid artery terminal branches to avoid bleeding that can make RLN visualization difficult. We use bipolar cautery to control the terminal branches, with tips cooled with a wet gauze between applications. As the remnant of the Berry’s ligament is transected, the retraction is reduced to avoid stretch injury to the RLN. Once the RLN branches are identified, they are traced inferiorly by dividing the remainder of the Berry’s suspensory ligament and releasing the RLN from the thyroid gland. The maximum extent of RLN dissection is 1 to 2 cm (Fig. 3c).

**Fig. 4:**
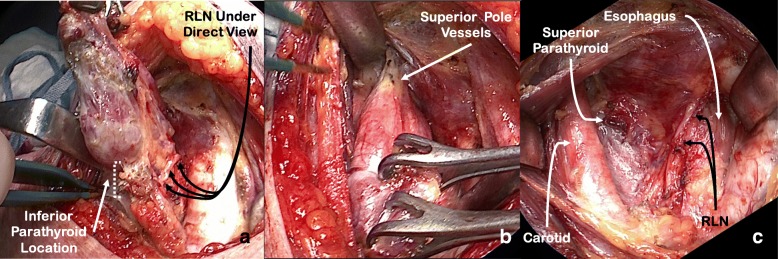
Capsular dissection with preservation of parathyroid tissue and ligation of superior pole vessels. **a** Identification of parathyroid glands while keeping the recurrent laryngeal nerve (RLN) under direct view; **b** Isolation and ligation of superior pole vessels; **c** Surgical bed after resection

4. Capsular dissection with preservation of parathyroid tissue and ligation of superior pole vessels (Fig. 4):

The thyroid gland is then swept medially, delivered through the wound, and retracted in an anterosuperior direction (Fig 4a). With the RLN in direct view, a capsular dissection is undertaken from inferior to superior direction. Elevation of the gland assists in exposure and isolation of the blood vessels feeding the parathyroid glands, helping their preservation under direct visualization (Fig. 4a).

The thyroid lobe remains pedicled on the superior pole vessels (Fig. 4b). Gentle retraction of the lobe inferiorly allows for their ligation and division, releasing the thyroid from the wound (Fig. 4c). Hemostasis is achieved and multi-layered closure is performed in the usual fashion.

## Results

Three hundred sixty-seven operations were eligible for inclusion. Twenty-five operations were excluded as either a central or a lateral neck dissection was performed concurrently. Out of 342 surgeries included in the analysis, 213 (62%) were hemi-, 91 (27%) were total, and 38 (11%) were completion thyroidectomies. The mean age of the patients was 50 years (range: 13–89 years), and 84% of patients were female.

The most common indications for a total thyroidectomy were papillary thyroid carcinoma (40%) and thyromegaly (27%) (Table 1). The most common indications for a hemithyroidectomy were nodules of intermediate suspicion for malignancy (54%) and thyromegaly (38%) (Table 1).

**Table 1:** Indications for surgery

	Total thyroidectomy *n* = 91	Hemi-thyroidectomy *n* = 213	Completion thyroidectomy *n* = 38	Total *n* = 342
Nodules requiring diagnosis				
Intermediate suspicion for malignancy	–	114 (54%)	2 (5%)	116 (34%)
High suspicion for malignancy	15 (16%)	2 (1%)	1 (3%)	18 (5%)
Thyromegaly				
Obstructive/Symptomatic	20 (22%)	73 (34%)	6 (16%)	99 (29%)
Substernal goiter	5 (5%)	8 (4%)	–	14 (4%)
Malignancy				
Papillary carcinoma	36 (40%)	2 (1%)	17 (45%)	55 (16%)
Follicular carcinoma	–	–	8 (21%)	8 (2%)
Poorly differentiated carcinoma	–	–	1 (3%)	1 (0.3%)
Lymphoma	–	–	1 (3%)	1 (0.3%)
Endocrinological diseases				
Graves’ disease	13 (14%)	1 (0.5%)	1 (3%)	15 (4%)
Hashimoto thyroiditis	1 (1%)	2 (1%)	–	3 (1%)
Other				
Symptomatic cyst	–	9 (4%)	–	9 (3%)
PET incidentaloma	1 (1%)	2 (1%)	–	3 (1%)

The final pathology results showed a variety of benign and malignant conditions (Table 2). The median surgical times for a total, hemi-, and completion thyroidectomy were 48 min (Interquartile range [IQR]: 40–60 min), 39 min (IQR: 33–47 min) and 40 min (IQR: 35–51 min) respectively (Table 3).

**Table 2:** Pathological diagnoses

	Total thyroidectomy (*n* = 91)	Hemi-thyroidectomy (*n* = 213)	Completion thyroidectomy (*n* = 38)	Total (*n* = 342)
Malignancies				
Papillary	50 (54%)	22 (10%)	12 (32%)	84 (25%)
Follicular	1 (1%)	11 (5%)	2 (5%)	14 (4%)
Poorly differentiated	1 (1%)	1 (0.5%)	–	2 (1%)
B-Cell lymphoma	–	1 (0.5%)	–	1 (0.3%)
Renal cell carcinoma	1 (1%)	–	–	1 (0.3%)
Goiters				
Multinodular goiter	16 (18%)	73 (34%)	9 (24%)	98 (29%)
Diffuse goiter	1 (1%)	5 (2%)	1 (3%)	7 (2%)
Endocrine diseases				
Graves’ disease	8 (9%)	1 (0.5%)	–	9 (3%)
Hashimoto’s thyroiditis	7 (8%)	10 (5%)	1 (3%)	18 (5%)
Other benign pathology				
Follicular adenoma	4 (4%)	65 (31%)	1 (3%)	70 (20%)
Thyroid cyst	–	5 (2%)	–	5 (2%)
Not otherwise specified	2 (2%)	19 (9%)	12 (32%)	33 (10%)

**Table 3:** Surgical times

	Total thyroidectomy Median (IQR)	Hemi-thyroidectomy Median (IQR)	Completion thyroidectomy Median (IQR)	Total Median (IQR)
All indications	48 min (40–60 min)	39 min (33–47 min)	40 min (25–93 min)	41 min (35–51 min)
Nodules requiring diagnosis	42 min (39–50 min)	38 min (32–44 min)	29 min (27–56 min)	39 min (32–45 min)
Thyromegaly				
Obstructive/Symptomatic	57 min (47–66 min)	41 min (35–50 min)	41 min (33–54 min)	44 min (37–53 min)
Substernal goiter	84 min (50–98 min)	60 min (52–65 min)	93 min	64 min (51–81 min)
Malignancy	47 min (40–53 min)	51 min (45–55 min)	39 min (36–46 min)	43 min (38–51 min)
Graves’ disease	55 min (43–63 min)	60 min	67 min	56 min (43–65 min)

*Abbreviations*: *IQR* interquartile range, *min* minutes

Complications experienced by the patients are summarized in Table 4. Out of 129 patients who underwent total or completion thyroidectomy, 17 (13%; 95% confidence interval [CI]: 8–20%) experienced transient and 4 (3%; 95% CI:1–8%) experienced permanent hypocalcemia. Out of 342 patients, 30 (9%; 95% CI: 6–12%) experienced temporary (i.e. 7% of nerves at risk), and 1 experienced permanent vocal cord palsy (0.3%; 95%CI: 0.01–2%). There were no instances of bilateral vocal cord palsy. It was unclear what caused the majority of temporary vocal cord palsies. In a minority of cases of temporary vocal cord palsy, the dictating surgeon commented on the difficulty due to inflammation and bleeding. In the single instance of permanent vocal cord paralysis, a RLN nerve branch was cut.

**Table 4:** Course in hospital and complications

	Total thyroidectomy *n* = 91	Hemi–thyroidectomy *n* = 213	Completion thyroidectomy *n* = 38	Total *n* = 342
Nights in hospital – median (Range)	1 (0–30)	0 (0–3)	1 (1–7)	1 (0 – 30)
Conversion to alternative approach – n (%)	2 (2%)	–	1 (3%)	3 (1%)
Complications – n (%)				
Transient hypocalcemia	16 (18%)	–	1 (3%)	17 (5%)
Permanent hypocalcemia	1 (1%)	–	2 (5%)	3 (1%)
Temporary vocal cord paresis/Paralysis	13 (14%)	14 (7%)	3 (8%)	30 (9%)
Permanent vocal cord paresis/Paralysis	1 (1%)	–	–	1 (0.3%)
Hematoma	1 (1%)	4 (2%)	1 (3%)	6 (2%)
Seroma	–	1 (0.5%)	–	
Wound infection	5 (6%)	3 (1%)	1 (2.6%)	9 (3%)
Subcutaneous emphysema	1 (1%)	2 (1%)	–	3 (1%)

In 3 patients (1%) a conversion to an alternative method of RLN dissection was needed, this was due to difficulty in controlling bleeding (2 cases) and dense scarring superior to Berry’s ligament (1 case).

## Discussion

In this study we introduce a systematic approach to dissecting the RLN at the cricothyroid junction, referred to here as the retrograde medial approach. The outcomes of this approach were investigated through a retrospective review of a single surgeon’s cohort of patients. The rates of transient and permanent vocal cord palsy were 9% (95% CI: 6–12%) and 0.3% (95%CI: 0.01–2%) respectively, while the rates of transient and permanent hypocalcemia were 13% (95% CI: 8–20%) and 3% (95% CI: 1–8%) respectively. The retrograde medial approach appears to be fast, with a median surgical time of 41 min.

Three previous studies have described using the superior approach to finding the RLN. While the authors of one study do not describe the specific technique [9], the authors of the other two studies followed the technique first described by Shindo et al [6, 10]. This technique involves releasing the superior pole, finding and releasing the tubercle of Zuckerkandl, retracting the gland medially, and then searching for the RLN as it courses towards the cricothyroid junction. Shindo et al. acknowledged the variability in the angle that the RLN takes as it approaches the cricothyroid junction and classified it into four categories. The authors also acknowledged that their technique is difficult in cases of nonrecurrent RLN, presence of large tubercle of Zuckerkandl, and extrathyroidal extension of cancer along the distal RLN segment.

In the presented retrograde medial approach, the RLN nerve is found early prior to the release of the superior pole and exploration of the thyroid’s lateral side. Given that the RLN is found superior to the Berry’s ligament, the variability in the angle that RLN takes in its approach has no impact on the dissection. Furthermore, the retrograde medial approach is preferential in cases with nonrecurrent RLN, large Zuckerkandl’s tubercle, and in the majority of cases with extrathyroidal extension of cancer along the distal RLN segment (with exception of cricothyroid junction involvement). We find the retrograde medial approach especially useful in cases of large goiters. Finally, an additional advantage of the retrograde medial approach is that no thyroid tissues is left unresected at the cricothyroid junction.

Some surgical situations make the use of retrograde medial approach difficult. The method relies on splitting the thyroid isthmus and identifying the inferior border of the cricopharyngeal muscle. If a surgeon is unable to complete these steps, either due to fear of tumor spillage with the isthmus division or extensive scarring, bleeding, or presence of tumor at the cricopharyngeus muscle or the cricothyroid junction, it is advisable that the lateral or inferior approach to the thyroidectomy is used.

With the use of the retrograde medial approach, the rates of permanent vocal cord palsy and hypocalcemia are similar to the rates reported with other superior approaches to finding the RLN; however, the rates of temporary vocal cord palsy and hypocalcemia appear higher. With regard to the permanent complications, in a study of 181 patients, Sykes et al. reported permanent vocal cord palsy and hypocalcemia rates of 0.4% and 2.2% respectively [10]. In a study of 67 patients, Veyseller et al. reported the corresponding rates to be 0 [9]. With regard to the transient complications, Sykes et al. reported a 2.2% rate of temporary vocal cord palsy [10]; whereas Veyseller et al. reported temporary vocal cord palsy and hypocalcemia rates of 0% and 8.3% respectively [9].

There are a number of possible reasons to why the rates of temporary complications were higher in the presented study than in the studies discussed above. First, given different sizes and compositions of populations studied, the difference could be secondary to sampling variability. Second, the difference might be due to different definitions of temporary complications. Specifically, compared to the mentioned studies we used a more liberal definition of hypocalcemia – requirement for calcium or vitamin D supplementation regardless of the reason. Finally, the technique described in this paper could be the reason for higher temporary complication rates. For example, the exclusive use of bipolar cautery could be responsible for transient RLN and parathyroid gland heat damage. Some surgeons might also suggest that traction at the Berry’s ligament transferred to the RLN might be responsible for higher transient vocal cord palsy rate. However, there is no tension on the nerve until the suspensory ligament is divided. Recognizing this is a point of importance and teaching. After division of the suspensory ligament the nerve is dissected out from top to bottom while on the slack at the fixation point at entrance to larynx.

Comparing the outcomes of the retrograde medial technique to the outcomes of the two other approaches (lateral and inferior) is difficult given that one approach is rarely used exclusively. Perhaps the best estimate of thyroidectomy complication rates comes from national studies. For example, the 2008 study of Scandinavian Quality Register for Thyroid and Parathyroid Surgery reported complication rates based on 3660 thyroidectomies [12]. In this report, the authors presented data on hypocalcemia defined identically to our study – the use of supplemental calcium and/or vitamin D at the first post-operative visit and 6 months after surgery. Using this definition, the rates of transient and permanent hypocalcemia in the Scandinavian study (17% and 6% respectively) appear similar to our study (13% and 3% respectively). With regard to vocal cord palsy, the authors of the Scandinavian study report temporary palsy rates lower than in our study (3.9% versus 9% respectively), but the permanent palsy rates appear similar to our study (0.9% versus 1% respectively).

With regard to surgical time, the authors of the studies described above do not report on the surgical speed. The speed of the described technique appears similar to the techniques described in recent meta-analyses and reviews of ultrasonic and electrothermal devices in thyroid surgery [13, 14]. For example, the authors of a recent meta-analysis concluded that a total thyroidectomy is performed faster with the use of Harmonic Focus® (Ethicon Inc., Cincinnati OH) than with the use of clips and ties [13]. The pooled average time for a total thyroidectomy using the Harmonic Focus® was 66 min and the average time for the conventional technique was 95 min [13]. In comparison, the average time for a total thyroidectomy in our series was 48 min.

Short operative time with the retrograde medial approach is possibly due to early RLN identification in a consistent location. The remaining dissection can then proceed quickly without concern of RLN injury. This is in contrast to the lateral approach in which complete RLN dissection is one of the last operating steps. We acknowledge that aside from the RLN dissection technique there might be other reasons for shorter operative time. These include the use of bipolar cautery in place of clips and ties and the surgical experience of the author.

The presented study has strengths. First, the described approach, while likely known to experienced thyroid surgeons, has not been previously reported in surgical literature. Second, while the presented technique is not meant to be prescriptive, prior to this study there has been no attempt to standardize identification of RLN at the cricothyroid junction. Finally, this study reports on the largest cohort of patients in literature for whom the superior approach to identifying RLN was used.

This series has limitations. First, a retrospective review might have resulted in missing data for complications. . Second, the study represents a single surgeon’s experience, possibly limiting the generalizability of the findings. Finally, our study excluded patients requiring lateral and central neck dissection; therefore, further investigation is required to determine the utility of the retrograde medial approach in such cases.

## Conclusion

A retrograde medial approach to identifying the RLN at the cricothyroid junction is described. This technique is useful in dissecting large goiters and when lateral RLN identification is difficult. In a tertiary endocrine head and neck practice, the routine use of the retrograde medial approach is safe and results in short operative time and a low conversion rate to other RLN dissection approaches.
